# Effects of inspiratory muscle training in adults with obesity and obstructive sleep apnea: a systematic review

**DOI:** 10.1007/s11325-026-03689-w

**Published:** 2026-04-25

**Authors:** Karina Abreu, Amanda Farias e Farias, Ananda Quaresma Nascimento, Alexandro Andrade, Darlan Laurício Matte

**Affiliations:** Center for Health and Sports Sciences (CEFID), Santa Catarina State University (UDESC), Florianópolis, Santa Catarina Brazil

**Keywords:** Obesity, Obstructive Sleep Apnea, Inspiratory Muscle Training, Sleep Disorders, Health-Related Quality of Life, Treatment Adherence, Sleep Fragmentation.

## Abstract

**Objective:**

To systematize and critically analyze scientific evidence on the effects of inspiratory muscle training (IMT) in adults with obesity and obstructive sleep apnea (OSA), focusing on outcomes related to OSA severity, respiratory muscle strength, respiratory function, cardiovascular parameters, sleep quality, and excessive daytime sleepiness.

**Methods:**

A systematic literature review was performed, in accordance with PRISMA 2020 guidelines. Searches were conducted in November 2025, across six databases (PubMed, Cochrane Library, SciELO, Scopus, Web of Science, and Embase). The review was registered in PROSPERO (CRD420251057891). Included studies were randomized controlled trials that evaluated the effects of IMT in adults with obesity and OSA.

**Results:**

Six studies were identified that investigated IMT in individuals with OSA and with an average body mass index BMI ≥ 30 kg/m². The primary outcomes assessed included the effects of IMT on maximal inspiratory pressure (MIP), the apnea-hypopnea index (AHI), cardiovascular parameters, excessive daytime sleepiness (EDS), sleep quality, neck and waist circumference, and functional capacity. The studies provided favorable evidence suggesting the use of IMT to improve these parameters, although some results showed inconsistencies.

**Conclusion:**

Although IMT is a low-cost, feasible, and home-based intervention, the current evidence is insufficient to support its recommendation as a standard therapeutic approach. Further high-quality randomized controlled trials with clearly defined obesity-based inclusion criteria and adequate follow-up are required to establish the effectiveness and clinical role of IMT as an adjunctive, non-pharmacological therapy in adults with obesity and OSA.

**Supplementary Information:**

The online version contains supplementary material available at 10.1007/s11325-026-03689-w.

## Introduction

Obesity is a multifactorial chronic disease characterized by excessive accumulation or abnormal distribution of body fat. Excess weight is traditionally classified using the body mass index (BMI) [1]. According to the World Health Organization (WHO), overweight in adults is defined as BMI ≥ 25 kg/m² and obesity as BMI ≥ 30 kg/m², which can be further subdivided into Class I (30–34.9 kg/m²), Class II (35–39.9 kg/m²), and Class III (≥ 40 kg/m²) [2]. In 2025, a new classification was proposed distinguishing a preclinical form, characterized by excess adiposity without functional impairment, from a clinical form, in which organ damage is already present. This approach recommends combining BMI with measures such as waist circumference and body composition to provide a more accurate assessment of health status [3]. Recent estimates indicate that more than 890 million adults are living with obesity, with continuous growth across all age groups and, particularly, in severe obesity [2, 4, 5].

Obesity is associated with several cardiometabolic comorbidities, including systemic arterial hypertension, type 2 diabetes mellitus, and cardiovascular diseases, and is the main modifiable risk factor for obstructive sleep apnea (OSA). The pathophysiology of OSA is complex and involves the interaction of anatomical and non-anatomical factors that compromise upper airway patency. Key mechanisms include cervical and pharyngeal fat deposition, reduced functional residual capacity, craniofacial structural alterations, fluid redistribution, and decreased neuromuscular responsiveness during sleep [6, 7]. Together, these factors predispose individuals to partial (hypopneas) or complete (apneas) upper airway collapse during inspiration, accompanied by hypoxemia, increased respiratory effort, and sleep fragmentation [8, 9].

The relationship between obesity and OSA appears to be bidirectional. While obesity increases the risk of developing OSA, the disorder itself contributes to weight gain through sleep disturbances, hormonal changes (leptin and ghrelin), systemic inflammation, and insulin resistance [10–12]. Clinically, individuals with OSA often present with fatigue, excessive daytime sleepiness, morning headaches, and metabolic, cardiovascular, and cognitive impairments, resulting in substantial reductions in quality of life and increased risk of accidents and morbidity [13].

OSA has a high prevalence, ranging from 9% to 38% in the general population, with higher rates among men, individuals with obesity, and older adults [14, 15]. Nearly 1 billion adults aged 30 to 69 may have OSA, most of whom remain undiagnosed [16]. Among women, prevalence rises sharply after menopause, even at BMI levels comparable to premenopausal women, due to reductions in estrogen and progesterone levels, which diminish upper airway muscle tone and increase susceptibility to pharyngeal collapse. These hormonal changes contribute to gender differences in OSA presentation and severity across the lifespan [17, 18].

Continuous positive airway pressure (CPAP) therapy is considered the gold-standard treatment for OSA [19], effectively reducing respiratory events and improving symptoms, especially in severe cases [16, 19]. However, adherence to CPAP is frequently low, with non-adherence rates ranging from 46% to 83% [20, 21]. Other therapeutic approaches, such as weight loss, physical exercise, oral appliances, positional therapy, orofacial myofunctional therapy, nasal valves, and bariatric surgery, may also benefit patients but often show limitations, variable responses, or challenges related to long-term maintenance [19, 22–27]. These adjunctive strategies not only reduce the apnea–hypopnea index (AHI) but may also improve metabolic health, reduce cardiovascular risk, and promote better sleep architecture.

In this context, complementary therapies such as inspiratory muscle training (IMT) have gained increasing interest. IMT involves resisted breathing exercises aimed at improving the strength and endurance of the inspiratory muscles and the oropharyngeal musculature [28], although IMT does not directly target the oropharyngeal musculature, this intervention affects oropharyngeal muscles through a mechanism of synergistic muscle activation and neural plasticity [29]. Preliminary evidence suggests that strengthening these muscles may reduce ventilatory effort, enhance upper airway stability, and decrease pharyngeal collapsibility during sleep, potentially lowering the AHI [13].

Despite this physiological plausibility, existing studies remain scarce, heterogeneous, and inconclusive regarding IMT’s effects on OSA severity, respiratory muscle function, and sleep quality, particularly in individuals with obesity [19]. The available literature is fragmented; as reviews on IMT in OSA generally do not stratify findings according to obesity status, whereas reviews focused on obesity exclude patients diagnosed with OSA. This gap is clinically relevant, since obesity imposes specific alterations in respiratory mechanics that may modify individual responses to IMT.

Unlike previous reviews, the present study specifically focuses on adults with concomitant obesity and OSA, emphasizing clinically relevant functional outcomes rather than solely polysomnographic indices, and aims to integrate these distinct bodies of literature.

Given this scenario, the following research question was proposed: In adults with obesity and a diagnosis of OSA, does IMT compared with no intervention, placebo, or other therapies improve outcomes related to OSA severity, respiratory muscle function, sleep quality, and other physiological and functional parameters (such as respiratory muscle strength, pulmonary function, cardiovascular measures, and exercise capacity)?

## Methods

This systematic review was conducted in accordance with the Preferred Reporting Items for Systematic Reviews and Meta-Analyses (PRISMA) guidelines [30]. The review was registered in the International Prospective Register of Systematic Reviews (PROSPERO) under registration number CRDxxx.

### Search strategy

A literature search was conducted in November 2025, using systematic searches in the PubMed/MEDLINE, Cochrane Library, SciELO, Scopus, Web of Science, and Embase databases. Primary search terms were prioritized in the title field, while secondary and tertiary search terms were included in all fields of each database; all descriptors used are described in Table 1.

**Table 1:** Search strategy for systematic review

Search terms	Descriptors
1. Sleep Apnea Obstructive	“Sleep Apnea, Obstructive” OR “obstructive sleep apnea” OR “sleep apnea” OR “sleep-disordered breathing” OR OSA OR OSAS OR “apnea hypopnea index” OR AHI
2. Inspiratory Muscle Training	“Breathing Exercises” OR “inspiratory muscle training” OR “inspiratory muscle strength training” OR IMT OR IMST OR “respiratory muscle training” OR “respiratory muscle strength” OR “inspiratory muscle strength”
Combination	#1 AND #2

The term “Obesity” was excluded from the search strategy in order to broaden the scope of the search, as its inclusion substantially reduced sensitivity, and obesity status was instead assessed during study selection.

The search and study selection processes were conducted independently by two reviewers. Discrepancies or disagreements between reviewers were resolved through consultation with a third reviewer.

## Eligibility criteria for studies

The following inclusion criteria were used to select eligible primary studies for this review: (1) randomized controlled trials (RCTs) involving IMT in individuals with age ≥ 18 years, with obesity (studies where the average BMI was ≥ 30 kg/m² were included) and OSA, (2) with variables related to OSA severity, respiratory muscle function, sleep quality, and physiological and functional parameters and (3) published in one of the following languages: English, Portuguese, or Spanish. Book chapters, conference abstracts, case reports, editorials, systematic reviews, meta-analyses, and opinion articles were excluded. Only studies that met all inclusion criteria were selected.

For this review, the eligibility criteria were based on the PICO strategy, as follows: P (Population): adults with BMI ≥ 30 kg/m² and OSA; I (Intervention): IMT; C (Comparison): no intervention, placebo, or other therapies; and O (Outcomes): outcomes related to OSA severity, respiratory muscle function, sleep quality, and physiological and functional parameters.

## Study selection and data extraction

The articles were independently screened by two reviewers (blinded to each other’s decisions), applying the inclusion criteria to the titles and abstracts, followed by full-text assessment. Any disagreements were resolved by consultation with a third reviewer.

All screening processes were conducted using Rayyan, a web and mobile application for systematic reviews [31].

For the analysis and discussion of the results, data were extracted from each article, including general information (author and publication date), study characteristics (method), participants (sample size, age, BMI, and AHI), intervention (equipment, intensity, frequency, duration, and number of sessions), control treatment, outcomes, and main findings.

## Methodological quality assessment

The methodological quality of the included studies was assessed using the PEDro Scale [32]. This scale evaluates methodological quality in RCTs and is composed of 11 items. The first item (eligibility criteria) is not included in the total score as it relates to external validity. Eight items are related to methodological quality (proper allocation, concealed allocation, baseline comparability, blinding of participants, therapists and assessors, adequate follow-up, and intention-to-treat analysis) and two items relate to statistical reporting (between-group comparisons, point estimates, and variability). According to the PEDro Scale, studies were categorized as low quality (up to 3 points), fair quality (4 or 5 points), or good quality (6 or more points).

## Risk of Bias assessment

The risk of bias assessment was independently conducted by two reviewers using the Cochrane Risk of Bias 2.0 (RoB 2) tool for RCTs, in accordance with the Cochrane Handbook guidelines [33]. Any discrepancies between reviewers were resolved through consensus.

Five domains were analyzed: bias arising from the randomization process, bias due to deviations from intended interventions, bias due to missing outcome data, bias in measurement of the outcome, and bias in selection of the reported result. For each domain, the studies were classified as having “low risk of bias,” “some concerns,” or “high risk of bias” [34].

The overall risk of bias classification was performed per outcome. Studies that presented a high risk in at least one domain, or “some concerns” in three or more domains, were classified as having a high overall risk. The remaining studies were categorized as having “some concerns” [34].

## Results

### Study selection

**Fig. 1:**
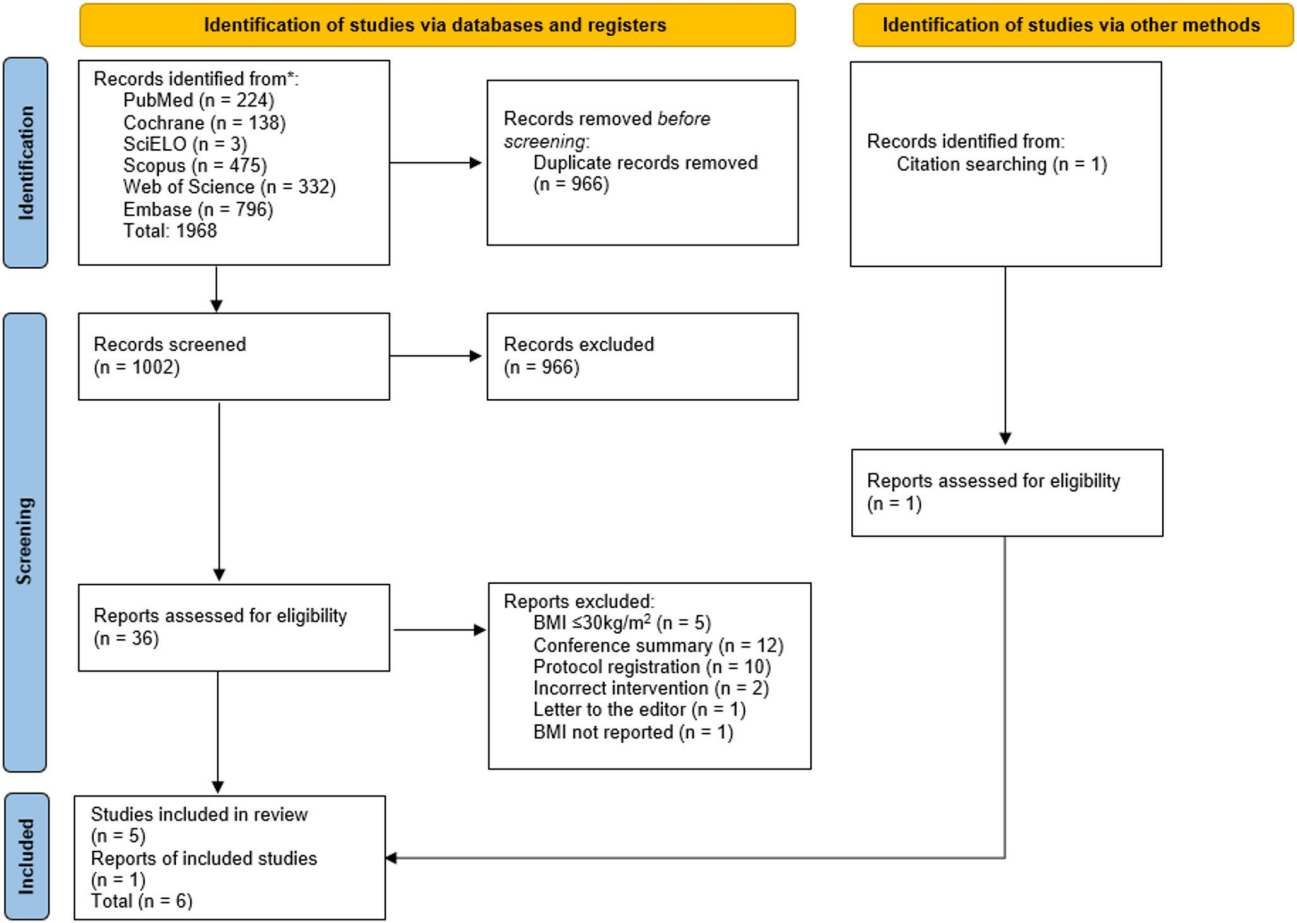
Flow diagram of the included studies. Source: Page, M.J. et al., 2021 (Page et al., 2021)

The initial search identified 1,968 potential studies. After removing 966 duplicates, 1,002 titles and abstracts were screened, resulting in the exclusion of 966 records. The full text of the remaining 36 studies was assessed for eligibility. Of these, 31 were excluded for the following reasons: five studies had a mean BMI ≤ 30 kg/m², 12 studies were conference summaries, ten were protocol registrations, two had an incorrect intervention, one was a letter to the editor, and one study was excluded for not reporting BMI (the corresponding author was contacted, but no response was received). Additionally, one study was identified through citation searching, assessed, and included. Thus, six studies met the eligibility criteria and were included in this systematic review (Fig. 1).

## Characteristics of the included studies

The characteristics of the included studies are shown in Table 2. The average age from 47.25 ± 7.32 to 69.7 ± 6.7 years and BMI from 30.0 ± 7.4 to 38.84 ± 3.4, only one study [35] strictly included participants with obesity. OSA severity ranged from mild to severe (AHI 5-≥30/hour) [36]. One study did not assess the severity of OSA, but rather the risk of OSA using the STOP-BANG scale [35].

**Table 2:** Characteristics of the studies included in the review

Author, year	Objective	SAMPLE	BMI (kg/m^2^) Obesity	Age (years)	AHI (events/hour)	Training	Results	
Stavrou et al. (2025)	To evaluate the effect of a 4-week RMT intervention versus CPAP on cardiorespiratory parameters and cognitive function in patients with OSA.	25 individuals with moderate to severe OSA (IG 14 and CG 14)	IG: 30.0 ± 7.4 CG: 32.5 ± 6.1 Grade I and II	IG: 51.3 ± 9.6 CG: 56.2 ± 5.9	IG: 22.4 ± 7.0 CG: 26.5 ± 5.5	Device: AirOFit PRO. IG IMT and EMT: Intensity: 80% of MIP and MEP. Progression: After 2 weeks, increase to 90% of MIP and MEP. Repetitions: 3 sets of 12 repetitions daily. Frequency and duration: One session per day for 4 weeks. CG: CPAP.	Increase in MIP: 95.1 vs. 105.9 ± 31.0 cmH_2_O, p 0.028) and MEP (108.7 ± 31.4 vs. 127.9 ± 46.7 cmH_2_O, p 0.022). Reduction in PSQI: 11.1 ± 5.8 vs. 9.1 ± 5.1, p 0.013. Significant improvement in MoCA: 23.4 ± 3.0 vs. 25.3 ± 2.4, *p* < 0.001). Regarding the STS: Improvement in mean arterial pressure at the end of the test (100.7 ± 4.9 vs. 91.9 ± 15.7 mmHg, *p* = 0.035), reduction in respiratory rate before (14.6 ± 4.3 vs. 10.9 ± 2.2 rpm, *p* < 0.001), at the end of the test (20.5 ± 4.1 vs. 16.9 ± 2.3 rpm, *p* < 0.001) and during recovery: 18.5 ± 4.8 vs. 14.4 ± 2.3 rpm, *p* < 0.001). Ventilatory efficiency improved significantly, evidenced by a reduction in VE/MVV (24.3 ± 7,8% vs. 20.1 ± 6.9%, *p* < 0,001) and resting VE/BSA (5.4 ± 1.8 vs. 4.3 ± 1.6, *p* = 0.024). Furthermore, there was a significant increase in oxygen breath (68.5 ± 27.6 vs. 80.7 ± 30.6 L/min/m^2^, *p* = 0.002), as well as in the PRQ both at rest (5.5 ± 2.5 vs. 7.0 ± 2.3, *p* < 0.001) and during the first minute of recovery (4.7 ± 1.8 vs. 5.9 ± 1.8, *p* < 0.001).	
Ferreira et al. (2022)	To determine clinical safety and cardiovascular, cardiac autonomic and inflammatory responses to a single session of IMT in OSA subjects.	40 individuals with moderate to severe OSA (IG 20 and CG 20)	IG: 34.0 ± 8.1 CG: 33.8 ± 6.9 Grade II and III	IG: 55.8 ± 9.1 CG: 55.4 ± 11.6	IG: 31.8 ± 18.2 CG: 30.9 ± 16.7	Device: Powerbreath^®^ Classiclight. IG IMT: Intensity: 70% of MIP. Progression: Not applicable. Repetitions: 3 sets of 30 breaths. Frequency and duration: Only one session. CG: IMT no load.	Heart rate decreased significantly (pre 79.5 ± 15.4, immediate post 77.7 ± 13.5 and post 1 h 71.2 ± 12.8 bpm, p 0.002). Heart rate variability: significant increase in RR interval (pre 837.5 ± 158.9, immediate post 877.6 ± 158.9 and after 1 h 905.6 ± 188.0 ms, *p* < 0.001), no difference in RR interval between groups (0.309).	
Nóbrega Junior et al. (2020)	To verify the effectiveness of 8 weeks of IMT on OSA severity and symptoms in individuals diagnosed with moderate or severe OSA.	16 individuals with moderate to severe OSA. (IG 8 and CG 8)	IG: 33.4 (30.3–34.5) CG: 32.7 (23.8–34.9) Grade I	IG: 58.6 ± 5.6 CG: 60.1 ± 2.7	IG: 31.7 ± 15.9 CG: 31.4 ± 20.8	Device: Powerbreath ^®^ Classic Light. IG IMT: Intensity: 50% of MIP for the first two weeks. Progression: 60% of MIP in weeks 3–4 and 75% of MIP in the last 4 weeks. Repetitions: 3 sets of 30 breaths. Frequency and duration: Twice a day, 7 days a week for 8 weeks. CG: IMT without load.	Reduction in AHI (31.7 ± 15.9 vs. 29.9 ± 15.8 events/h; *p* < 0.001), in Berlin scores (2.6 ± 0.5 vs. 1.2 ± 0.5; *p* = 0.016), PSQI (7.2 ± 3.6 vs. 3.7 ± 1.3; *p* = 0.008), ESS (12.5 ± 4.0 vs. 7.7 ± 3.0; *p* = 0.008) and increase in MIP (83.6 ± 26.5 and 127.9 ± 32.5 cmH_2_O; *p* = 0.010).	
Erturk et al. (2020)	To compare the effects of IMT and OE on outcomes relevant to OSA patients.	41 individuals with mild, moderate, and severe OSA. (IMT 15, OE 14 and CG 12)	IG IMT: 31.00 ± 5.42 IG OE: 31.36 ± 3.84 CG: 32.06 ± 3.69 Grade I and II	IG IMT: 49.66 ± 9.08 IG OE: 53.71 ± 7.08 CG: 47.25 ± 7.32	IG IMT: 30.00 ± 19.33 IG OE: 42.6 ± 27.1 CG: 38.70 ± 23.98	Device: Threshold. IG IMT: Intensity: 30% of MIP. Progression: Load was adjusted weekly to a new MIP. Breaths: 8 breaths, after 4–5 controlled breaths for 15 min. Frequency and duration: 7 days a week for 6 weeks. IG: OE; CG: No program.	Reduction in the risk of OSA and snoring frequency (Berlin): (pre 2.8 ± 0.77 vs. post 1.06 ± 1.22, *p* < 0.001 and pre 3.2 ± 1.37 vs. post 0.93 ± 1.09, *p* < 0.001), in fatigue severity (FSS) (pre 4.31 ± 1.62 vs. post 2.77 ± 1.67, *p* < 0.001) and PSQI (pre 7.46 ± 4.15 vs. post 2.5 ± 1.7, *p* < 0.001). Reduction in neck circumference (pre 38.66 ± 3.30 vs. post 38.1 ± 3.54 cm, *p* = 0.008) and waist circumference (pre 97.23 ± 9.68 vs. post 94.13 ± 9.63 cm, *p* < 0.001), improvement in respiratory muscle strength (MIP pre 80.93 ± 16.93 vs. post 126.46 ± 15.62 cmH_2_O, *p* < 0.001), MEP pre 120.46 ± 21.32 vs. post 160.26 ± 28.56 cmH_2_O, *p* < 0.001). Reduction in ESS (pre 8.93 ± 4.41 vs. post 3.06 ± 3.49, *p* < 0.001), improvement in FOSQ (pre 10.4 ± 2.88 vs. post 14.65 ± 1.27, *p* < 0.001).	
Andhare; Yeole; Fakhri (2020)	To check the effect of IMT in OSA on obese people.	100 individuals with moderate to severe OSA. (IG 50 and CG 50)	IG: 38.84 ± 3.4 CG:36.78 ± 4.12 Grade II and III	IG: 51.22 ± 5.43 CG: 50.35 ± 7.06	STOP-BANG: IG: 5.22 ± 0.78 CG:5.20 ± 1.29 p0.98	Device: Threshold IMT. IG IMT: Intensity: Minimal load. Progression: Gradually up to 60–80% of 1 RM. Repetitions: 3–4 sets of 5 min. Frequency and duration: Once a day, 3 times per week for 4 weeks. CG: Conventional breathing exercises.	Reduction in score STOP-BANG: (pre 5.22 ± 0.78 vs. post 4.08 ± 1.45, p 0.0001), improvement in PSQI (pre 10.64 ± 1.63 vs. post 9.12 ± 1.59, p 0.0001) and increase in respiratory parameters (1RM: pre 23.12 ± 3.108 vs. post 25.44 ± 2.837 cmH_2_O, p 0.0002 vs. PEFR pre 227.6 ± 32.6 vs. post 249.2 ± 39.4 L/min, p0.0408).	
Ramos-Barrera; DeLucia; Bailey (2020)	To assess the effects of IMST on cardiovascular parameters in older, overweight adults diagnosed with moderate and severe OSA.	25 individuals at high risk for OSA. (IG 15 and CG 10)	IG: 30.7 ± 6.2 CG: 31.3 ± 6.5 Grade I	IG: 65.9 ± 6.0 CG: 69.7 ± 6.7	IG: 22.9 ± 11.0 CG: 26.2 ± 13.5	Device: POWERbreathe (K3 Series). IG IMT: Intensity: 75% of MIP Progression: The target pressure was assessed at the end of each training week. Repetitions: 5 sets of 6 breaths. Frequency and duration: 7 days a week, for 6 weeks. CG: IMT with 15% of MIP.	Significant increase in MIP (82.6 ± 12.5 vs. 116.5 ± 13.6 cmH₂O, *p* < 0.001). Cardiovascular measurements: significant reduction in SBP, DBP, and MAP (SBP: -8.82 ± 4.98; DBP: -4.69 ± 2.81; and MAP: -6.06 ± 1.03 mmHg; *p* < 0.002), ambulatory blood pressure monitoring (nighttime SBP 141.6 ± 18.9 vs. 129.6 ± 15.7 mmHg, *p* < 0.01), muscle sympathetic nervous activity MSNA: significant reduction in burst incidence (91.7 ± 6.7 vs. 83.5 ± 9.3 bursts/100 heartbeats, *p* < 0.01), and burst frequency (53.7 ± 7.0 vs. 46.7 ± 8.0 bursts/min, *p* < 0.002). < 0.01).	

IMT interventions [37–41] used loads of 30%–80% maximal inspiratory pressure (MIP), either as a standalone therapy [35, 37, 39, 41] or combined with expiratory muscle training (EMT) [40], or oropharyngeal exercises [38].

The control groups varied between CPAP [40], sham IMT [37, 41], 15cmH₂O loads [39], no physiotherapy [38] and conventional respiratory exercises [35]. Protocol duration and frequency ranged from single sessions to daily training for three months [37–41].

Data presented as mean ± standard deviation. Legend: BMI: Body Mass Index (kg/m^2^); OSA: Obstructive Sleep Apnea; IG: Intervention Group; CG: Control Group; RMT: Respiratory Muscle Training; IMT: Inspiratory Muscle Training; EMT: Expiratory Muscle Training; IMST: Inspiratory Muscle Strength Training; CPAP: Continuous Positive Airway Pressure; MIP: Maximal Inspiratory Pressure; MEP: Maximal Expiratory Pressure; AHI: Apnea-Hypopnea Index; MoCA: Montreal Cognitive Assessment; VE/MVV: Ratio between minute ventilation and maximal voluntary ventilation; VE/BSA: Ratio of minute ventilation (L/min) to the individual’s body surface area (m^2^), expressed in L/min/m^2^; PRQ: Pulse-Respiration Quotient; ESS: Epworth Sleepiness Scale; PSQI: Pittsburgh Sleep Quality Index; FSS: Fatigue Severity Scale; FOSQ: Functional Outcomes of Sleep Questionnaire; 1RM: 1 Repetition Maximum; PEFR: Peak Expiratory Flow Rate; OE: Oropharyngeal Exercises; Berlin: Berlin Questionnaire; STOP-BANG: STOP-BANG scale; SBP: Systolic Blood Pressure; DBP: Diastolic Blood Pressure; MAP: Mean Arterial Pressure; MSNA: Muscle Sympathetic Nervous Activity.

### Main outcomes

Table 3 summarizes the main outcomes: AHI [37–39], respiratory muscle strength [35, 37–40], functional capacity (39,41), pulmonary function [35, 39, 40], cardiovascular measurements [39–41], sleep quality [35, 37–40], daytime sleepiness [37, 38], body composition [38, 40], and cognitive function [40].

**Table 3:** Main results of the included studies. Legend: (=) no statistically significant changes; (↑) significant increase in the variable; (↓) significant reduction in the variable; (NE) not evaluated. RCT: Randomized Clinical Trial; AHI: Apnea-Hypopnea Index; PSQI: Pittsburgh Sleep Quality Index; ESS: Epworth Sleepiness Scale; Berlin: Berlin Questionnaire; MoCA: Montreal Cognitive Assessment; RMT: Respiratory Muscle Training; CPAP: Continuous Positive Airway Pressure; OSA: Obstructive Sleep Apnea; IMT: Inspiratory Muscle Training

Authors	Study Design	Objective	AHI	Respiratory muscle strength	Pulmonary function	Functional capacity	Cardiovascular measurements	Cervical circumference	MoCA	PSQI	ESS	Berlin
Stavrou et al. (2025)	RCT	To evaluate the effect of a 4-week RMT intervention versus CPAP on cardiorespiratory parameters and cognitive function in patients with OSA.	NE	↑	=	↑	↓	=	↑	↓	NE	NE
Ferreira et al. (2022)	RCT	To determine clinical safety and cardiovascular, cardiac autonomic and inflammatory responses to a single session of IMT in OSA subjects.	NE	NE	NE	NE	↓	NE	NE	NE	NE	NE
Nóbrega Junior et al. (2020)	RCT	To verify the effectiveness of 8 weeks of IMT on the severity and symptoms of OSA in subjects diagnosed with moderate or severe OSA.	↓	↑	NE	NE	NE	NE	NE	↓	↓	↓
Erturk et al. (2020)	RCT	To compare the effectiveness of IMT and oropharyngeal exercise in patients with OSAS in terms of disease severity, respiratory muscle strength, exercise capacity, severity and frequency of snoring, daytime sleepiness, effects of sleepiness on daily life, and sleep quality.	=	↑	NE	=	NE	↓	NE	↓	↓	↓
Andhare; Yeole; Fakhri. (2020)	RCT	To check the effect of IMT in OSA in obese people.	NE	↑	↑	NE	NE	NE	NE	↓	NE	NE
Ramos-Barrera; Delucia; Bailey (2020)	RCT	To assess the effects of IMST on cardiovascular parameters in older, overweight adults diagnosed with moderate and severe OSA.	=	↑	=	NE	↓	NE	NE	=	NE	NE

### Assessments and devices

AHI was assessed through polysomnography [37–39]. Screening and the identification of risk factors, including the presence, frequency, and intensity of snoring, were performed using the Berlin Questionnaire [42], while the STOP-Bang was employed for OSA screening and risk estimation [35]. Snoring frequency was assessed using the snoring-related items embedded within the STOP-Bang scale and Berlin questionnaire, based on self-reported responses, without objective acoustic measurement.

MIP was measured with a manovacuometer [43]; POWERbreathe KH2 [44]; and Airofit PRO [40]. Functional capacity was assessed by the six-minute walk test (6MWT), which is commonly used to measure functional exercise capacity, evaluate prognosis, and assess treatment response in a wide range of chronic respiratory diseases [45] and 30-second Sit-to-Stand (30s StS). The 30s STS is used to evaluate the physical performance and functional capacity of the lower limbs [46].

Pulmonary function was evaluated using spirometry [47]. Body composition was estimated using bioelectrical impedance analysis [48]. Cardiovascular parameters were assessed using a blood pressure monitor [49], pulse transit time technology (integrating electrocardiogram and pulse oximetry) to preserve sleep architecture [50], a heart rate monitor [51], and a cardiopulmonary exercise testing (CPET) system with breath-by-breath gas analysis [28].

Sleep quality was assessed using the Pittsburgh Sleep Quality Index (PSQI). A total score > 5 on the PSQI indicates poor sleep quality [52]. The Epworth Sleepiness Scale (ESS) was used to evaluate daytime sleepiness. Scores above 10 suggest the diagnosis of excessive daytime sleepiness [52]. Cognitive assessment was performed using the Montreal Cognitive Assessment (MoCA), employed to detect cognitive impairment associated with sleep disorders. This instrument evaluates several cognitive domains, including executive function, visuospatial abilities, memory, and attention, with a total score ranging from 0 to 30 [53].

### Methodological quality assessment

Regarding the methodological quality of the included studies, four studies showed fair quality, with PEDro scores of 4/10 [35], and 5/10 [38–40], while the remaining studies showed good quality, with PEDro scores of 6/10 and 8/10, respectively [37, 41]. The methodological quality of the studies is presented in Supplement 1.

### Risk of bias assessment

According to the RoB 2 tool (Supplement 2), no study presented a low overall risk of bias; three showed “some concerns” [37, 38, 41], and three were “high risk” [35, 39, 40]. High risk in the randomization process [35, 39, 40] was the primary bias source, though two studies [37, 41] maintained low risk.

All studies showed low risk for deviations from intended interventions. Missing outcome data was mostly low risk, except for one study [37]. Regarding outcome measurement, two studies [37, 39] showed low risk, while others had “some concerns” due to lack of blinding. All studies were rated as “some concerns” in the selection of reported results, primarily due to the absence of pre-registered protocols.

### Results synthesis

Five studies [35, 37–40] showed that IMT significantly increased the inspiratory muscle strength of participants in the intervention groups. A statistically significant reduction in AHI was observed in one study [37]. However, the studies by Erturk et al. (2020) [38] and Ramos-Barrera; Delucia and Bailey (2020) [39] did not find significant differences in AHI after the IMT intervention.

It is important to note that STOP-BANG and Berlin questionnaires are screening tools and do not provide diagnostic confirmation of OSA severity. Compared to the Berlin Questionnaire, the STOP-Bang demonstrates higher diagnostic sensitivity, making it superior for detecting all levels of OSA severity in clinical settings [54]. Nonetheless, there was a reduction in OSA-related symptoms as measured by changes in STOP-BANG scale scores [35] and the Berlin questionnaire [37, 38].

Aspects related to daytime sleepiness showed a statistically significant reduction in ESS scores in the IMT groups [37, 38], sleep quality significantly improved in participants undergoing IMT according to the PSQI [35, 37, 38, 40], whereas the study by [39] did not show a significant improvement in PSQI. In the study by Erturk et al. (2020) (39), there was a statistically significant reduction in total Fatigue Severity Scale (FSS) scores. Body composition showed a significant decrease in neck and waist circumferences [38], contrasting with the findings of [40], where neck circumference remained unchanged after IMT. Regarding cognitive function [40], reported a significant increase in total MoCA scores, with improvements being most prominent in the attention and working memory domains.

Regarding functional capacity, no significant changes were observed in the distance covered in the 6MWT for the IMT and RMT groups [38]. Similarly, no statistical difference was found in the number of repetitions in the 30s STS test following the IMT intervention; however, a significant improvement in cardiorespiratory parameters was noted during the test [40].

Regarding lung function, no significant improvements were found following IMT in the following parameters: FEV1 (forced expiratory volume in one second), FVC (forced vital capacity), FIV1 (forced inspiratory volume in one second), FIVC (forced inspiratory vital capacity), FEV1/FVC and FIV1/FIVC ratios, PEF (peak expiratory flow), and PIF (peak inspiratory flow) [39]. Similarly, no statistical differences were observed for FEV1, DLCO (diffusing capacity for carbon monoxide), and TLC (total lung capacity) in the study by [40]. Nevertheless, in the study by Andhare; Yeole and Fakhri (2020) [35], IMT increased PEF.

Regarding cardiovascular and ventilatory outcomes, Stavrou et al. (2025) [40] demonstrated that IMT significantly enhances cardiorespiratory efficiency. During functional testing, a reduction in mean arterial pressure (MAP) was observed, alongside marked improvements in ventilatory efficiency. Specifically, there was a significant decrease in the VE/MVV ratio and resting VE/BSA. Furthermore, participants showed an increase in oxygen uptake efficiency and a higher Respiratory Quotient (RQ) at rest and during the first minute of recovery. Consistent with these findings, Ramos-Barreira; DeLucia and Bailey (2020) [39] observed substantial clinical reductions in systolic blood pressure (SBP), diastolic blood pressure (DBP), and mean arterial pressures. Furthermore, ambulatory blood pressure monitoring (ABPM) in the same study revealed a significant drop in nocturnal SBP, which was associated with a decrease in muscle sympathetic nerve activity (MSNA), evidenced by a reduction in both burst incidence and frequency. In contrast, Ferreira et al. (2021) [41] did not find immediate or short-term (1-hour post-session) changes in SBP or DBP. However, they noted a significant reduction in heart rate and an improvement in heart rate variability, characterized by a significant increase in the RR interval, suggesting an enhancement in autonomic modulation following IMT. The associations between IMT with sleep quality and respiratory muscle strength are presented in Table 4.

**Table 4:** Main findings of the studies on the associations of IMT with sleep quality and respiratory muscle strength in individuals with obesity and OSA. Legend: (↑) Association of IMT with sleep quality or respiratory muscle strength; (↓) No association of IMT with sleep quality or respiratory muscle strength; (NE) Not Evaluated

Study	*N*	Results	Association -IMT with sleep quality	Association -IMT with respiratory muscle strength
Stavrou et al. (2025)	25	IMT was associated with improved sleep quality (11.1 ± 5.8 vs. 9.1 ± 5.1; *p* = 0.013) and increased inspiratory muscle strength (95.1 ± 23.1 vs. 105.9 ± 31.0; *p* = 0.028).	↑	↑
Ferreira et al. (2022)	40	Sleep quality and respiratory muscle strength were not measured.	NE	NE
Nóbrega Junior et al. (2020)	16	IMT was associated with improved sleep quality (7.2 ± 3.6 vs. 3.7 ± 1.3; *p* = 0.008) and increased inspiratory muscle strength (83.6 ± 26.5 vs. 127.9 ± 32.5; *p* = 0.010).	↑	↑
Erturk et al. (2020)	41	IMT was associated with improved sleep quality (7.46 ± 4.15 vs. 2.5 ± 1.7; *p* < 0.001) and increased inspiratory (80.93 ± 16.93 vs. 126.46 ± 15.62; *p* < 0.001) and expiratory muscle strength (120.46 ± 21.32 vs. 160.26 ± 28.56; *p* < 0.001).	↑	↑
Ramos-Barrera; Delucia; Bailey (2020)	25	IMT was not associated with improved sleep quality (9.0 ± 5.0 vs. 8.6 ± 4.0; *p* > 0.1) and was associated with increased inspiratory muscle strength (82.6 ± 12.5 vs. 116.5 ± 13.6; *p* < 0.001).	↓	↑
Andhare; Yeole; Fakhri (2020)	100	IMT was associated with improved sleep quality (10.64 ± 1.63 vs. 9.12 ± 1.59; *p* = 0.0001). Respiratory muscle strength was not measured.	↑	NE

## Discussion

The current systematic review mapped studies that used IMT in individuals with OSA and BMI suggestive of obesity and showed improvements in respiratory muscle strength, daytime sleepiness, sleep quality, and cardiovascular parameters. The methodological quality of the analyzed studies ranged from fair to good, according to the evaluation by the Physiotherapy Evidence Database – PEDro Scale. The risk of bias ranged from some concerns to high overall risk of bias according to assessment using RoB 2. The results should be interpreted with caution due to the high heterogeneity in the included studies. The findings of this study are presented in Supplement 3.

### Respiratory muscle strength

In individuals with obesity, there is fat accumulation in regions such as the neck, thorax, and soft tissues, which can cause narrowing of the upper airway (UA), both relatively and absolutely. This narrowing significantly contributes to UA collapse during sleep [55, 56]. Azeredo et al. (2022) [13] suggest that IMT can improve the function of primary and accessory respiratory muscles, reducing the propensity for upper airway collapse during sleep.

As expected by the principle of training specificity, the five studies that assessed inspiratory muscle strength observed significant increases in MIP and the one-repetition maximum test (1RM). This is because IMT employs devices that provide flow-independent resistance, respecting the principles of physical training [28].

These findings are consistent with the results of [57], who attribute the increase in MIP in individuals with obesity to mechanisms such as diaphragm hypertrophy, increased proportion and size of type II muscle fibers, attenuation of the respiratory metaboreflex, and improved efficiency of respiratory muscle work.

### Apnea-hypopnea index

One study reported a significant reduction in AHI [37]. Consistent with this finding, a significant reduction in AHI was observed following an IMT protocol in individuals with moderate to severe OSA [13, 28]. On the other hand, two studies [38, 39] did not identify statistically significant improvements. This divergence may be related to a dose-response relationship involving training volume, intensity, and intervention duration. While Nóbrega-Júnior et al. (2020) [37] achieved significant reductions in AHI using a high-volume protocol (180 breaths daily for 8 weeks), Azeredo et al. (2022) [13] demonstrated that a lower daily volume (30 breaths) could still be effective if sustained over a longer period of 12 weeks. In contrast, the protocols by Erturk et al. (2020) and Ramos-Barrera; Delucia; Bailey (2020) [38, 39] failed to show significant changes in AHI, likely due to their shorter duration (6 weeks) combined with either low training intensity (30% MIP) [38] or low daily volume (30 breaths) [39]. These findings indicate that to achieve a clinically relevant reduction in the frequency of respiratory events, IMT protocols may need to reach a specific physiological threshold, either through high daily repetition counts or extended intervention periods.

### Excessive daytime sleepiness and sleep quality

Previous studies have demonstrated a strong association between IMT and improvements in excessive daytime sleepiness (EDS) [28, 58], which is reinforced by the findings of this review. The two studies that evaluated EDS reported significant improvements in this outcome and in aspects of general functional status. In the study by Nóbrega-Júnior et al. (2020) [37], improvement in EDS was also observed in the placebo group. This may be related to the fact that both groups participated in a structured intervention, which increased the level of physical activity in previously sedentary individuals, contributing to reduced sleepiness [37].

Regarding sleep quality, five studies used the Pittsburgh Sleep Quality Index (PSQI). Of these, four demonstrated a significant improvement in the total score, indicating a transition to better overall sleep quality. These findings are consistent with the literature [13, 59], who also reported a significant improvement in the global PSQI score. Only the study by Ramos-Barrera; Delucia and Bailey (2020) [39] did not show significant changes in sleep quality, which may be explained by baseline PSQI score and the reduced sample size. This could have occurred due to the reduced sample size, limiting the representativeness of the studied population.

### Pulmonary function

Only two studies evaluated pulmonary function before and after the IMT intervention and did not find significant improvements in FEV1, FVC, FIV1, FIVC, FEV1/FVC and FIV1/FIVC ratios, PEF, PIF, DLCO and TLC [39, 40]. This lack of effect may be attributed to the fact that participants had preserved pulmonary function at baseline [40]. Although specific baseline pulmonary function data were not presented [39].

Similarly, Nóbrega-Júnior et al. (2020) [37] also observed FEV1 and FVC values ≥ 80% of the predicted values for the Brazilian adult population. These findings corroborate those of Souza et al. (2018) [59], who also identified preserved pulmonary function at baseline, which may justify the absence of changes after IMT.

On the other hand, Andhare; Yeole and Fakhri (2020) [35] reported baseline peak expiratory flow (PEF) values below the predicted levels for adults, with significant improvement after the intervention, suggesting that individuals with impaired pulmonary function may benefit more from IMT. The study by Lin; Chiang and Ong (2020) [28] also demonstrated improvements in FVC and FEV6 (forced expiratory volume in six seconds) after IMT, even in participants with baseline values within normal limits.

Furthermore, Ayiky et al. (2024) [60] reported significant reductions in FVC, FEV1, PEF, TLC (Total Lung Capacity), and FRC (Functional Residual Capacity) in individuals with obesity and moderate to severe OSA compared to non-obese groups. Thus, the absence of changes in pulmonary functional parameters in some studies may be related to the lower BMI and lower OSA severity of their participants when compared to samples with greater respiratory impairment, such as those reported by Ayik et al. (2024) [60], where the mean BMI was 39.26 ± 5.80 kg/m².

### Cardiovascular measurements

Significant improvements in cardiovascular parameters were observed across the reviewed studies. These findings suggest that IMT acts as a potent modulator of the autonomic nervous system. The reduction in blood pressure and muscle sympathetic nerve activity (MSNA) reported by Ramos-Barrera; DeLucia; Bailey (2020) [39] is corroborated by the findings of Mello et al. (2012) [61], who demonstrated that 12 weeks of IMT significantly reduce both MSNA burst frequency and incidence. This physiological adaptation is likely driven by the attenuation of the inspiratory muscle metaboreflex; as inspiratory muscles become stronger and more efficient, sympathetic-mediated peripheral vasoconstriction is reduced [61].

Furthermore, the meta-analysis by Zheng et al. (2023) [62] reinforces these findings by demonstrating significant reductions in SBP and DBP, as well as resting heart rate, following IMT. Zheng et al. (2023) [62] suggest that these benefits stem from enhanced cardiopulmonary coupling and increased vagal activity. Consequently, the autonomic improvement and the increase in heart rate variability observed by Ferreira et al. (2022) [41], coupled with the cardiorespiratory enhancement found by Stavrou et al. (2025) [40], suggest that IMT promotes a systemic response that favors hemodynamic stability in patients with respiratory and cardiovascular impairment.

### Aerobic and functional capacity

Regarding functional capacity, there were no differences in performance on the 6MWT and 30s STS functionality tests. This lack of effect may be explained by the fact that the training focused on respiratory and pharyngeal muscles, without including aerobic or peripheral muscle exercises, and the improvement in exercise capacity is directly related to the volume of muscle mass involved in the effort [38].

Similarly, in the study by Vivodtzev et al. (2018) [58], participants were divided into three groups: cycling, cycling combined with NIV, and cycling combined with respiratory muscle training (RMT). All three groups showed improvement in the 6MWT distance, with no statistical difference between the groups after three months of training. Therefore, RMT did not lead to additional improvement in the 6MWT compared to the other intervention groups. These results suggest that the effectiveness of IMT in functional measures may depend on the concomitant inclusion of aerobic physical exercise.

#### Body composition

Body composition was addressed in two studies. Only one study [38] demonstrated a significant reduction in neck and waist circumference after the IMT protocol. However, the control group also showed a reduction in neck circumference, which limits the interpretation of the results. Additionally, the authors highlight that the baseline neck and waist circumference values were lower in the IMT group compared to the control group, which may have influenced the magnitude of the observed reduction.

These findings are similar to those reported by Ieto et al. (2015) [63] who applied a training protocol with oropharyngeal exercises targeting the genioglossus and pharyngeal muscles. The protocol consisted of 8-minute sets, three times a day, for three months. At the end of the intervention, a significant reduction in neck circumference was observed, indicating that localized muscle training can influence specific anthropometric measures associated with OSA [63].

### Cognitive function

The improvement in cognitive function, evidenced by the increase in MoCA scores and improvement in the attention and working memory domains [40], is linked to the reduction of excessive daytime sleepiness and the improvement in sleep quality (38,39). The restoration of restorative sleep, demonstrated by significant improvements in PSQI and ESS scores [35, 37, 38], functions as a supporting mechanism for cortical recovery and increased vigilance [37, 40]. Although increased general physical activity may contribute to reduced sleepiness [37], the specific benefits of IMT suggest that respiratory strengthening promotes hemodynamic stability and ventilatory efficiency [39, 40], which directly improves neuropsychological performance [40].

#### Strengths and innovations of the study

This systematic review addresses an important gap in the literature by evaluating the effects of IMT in individuals with OSA and BMI ≥ 30 kg/m². Although obesity and OSA are closely linked and highly prevalent conditions, the role of IMT as a therapeutic strategy in this specific population remains underexplored. By focusing on studies that included predominantly obese adults, this review offers a more nuanced perspective than previous broader reviews that did not account for BMI. A further strength lies in highlighting IMT as a potentially accessible, low-cost, and home-based intervention, an important consideration given the challenges of adherence associated with standard therapies, such as CPAP. This reinforces the clinical relevance of investigating alternative or adjunct approaches for individuals who may not tolerate or benefit fully from conventional treatments.

#### Clinical implications, suggestions for professional practice and new protocols

##### Incorporation of IMT as a complementary therapy

Inspiratory muscle training can be considered an adjuvant therapy in the management of obstructive sleep apnea in individuals with obesity, especially for patients with low adherence to CPAP. The practice can be performed at home, facilitating adherence and offering an accessible, low-cost complementary intervention.

##### Proper training and monitoring

Healthcare professionals, such as respiratory physiotherapists, should be trained to implement IMT protocols based on the individual needs of patients, and to monitor the effects on respiratory function and associated clinical outcomes, such as sleep quality and MIP.

##### Individualization of treatment

Considering the variations in study results, it is essential that the application of IMT be individualized, considering factors such as degree of obesity, and severity of OSA and associated comorbidities, in order to optimize clinical outcomes and improve quality of life in patients.

##### Patient education and treatment adherence

Educating patients about the benefits of IMT and its role as part of OSA management can increase adherence to treatment. It is crucial that patients understand the importance of practicing IMT regularly to improve respiratory function and reduce OSA symptoms.

#### Limitations and future directions

The included studies showed substantial clinical and methodological heterogeneity. Notably, only one trial applied inclusion criteria specifically targeting individuals with obesity. Therefore, studies were included based on mean BMI, implying that some participants may not have been obese, which limits the generalizability of the findings.

In addition, the methodological quality of the included studies was heterogeneous, with only two classified as good quality and several presenting a high risk of bias. This heterogeneity, together with differences in intervention protocols and outcome measures, precluded a meta-analysis and limits the strength of the conclusions.

Other limitations include small sample sizes and the absence of follow-up assessments, preventing conclusions regarding the long-term effects of IMT.

Future RCTs with greater methodological rigor are warranted. These should include clearly defined obesity-based eligibility criteria, standardized IMT protocols, adequate sample sizes, and longer follow-up periods to clarify the role of IMT as a complementary or adjunct non-pharmacological therapy, rather than a replacement for CPAP, in adults with obesity and obstructive sleep apnea.

## Conclusion

The present study synthesized and critically appraised the available scientific evidence on the effects of IMT in adults with obesity and OSA. The analysis revealed a scarcity of studies specifically targeting individuals with obesity and OSA, with only one trial recruiting exclusively obese participants, which limits the generalizability of the findings.

Despite these limitations, preliminary evidence suggests that IMT may provide benefits for adults with OSA and an elevated BMI, particularly by improving inspiratory muscle strength, reducing excessive daytime sleepiness, enhancing sleep quality, and with potential improvements in cardiovascular parameters. However, findings related to pulmonary function, AHI, and body composition remain inconsistent, and substantial heterogeneity was observed in intervention protocols and outcome measures.

Although IMT is a low-cost, feasible, and home-based intervention, the current evidence is insufficient to support its recommendation as a standard therapeutic approach. Further high-quality RCTs with clearly defined obesity-based inclusion criteria and adequate follow-up are required to establish the effectiveness and clinical role of IMT as an adjunctive, non-pharmacological therapy in adults with obesity and OSA.

## Electronic supplementary material

Below is the link to the electronic supplementary material.

Supplementary Material 1 (DOCX 53.5 KB)

Supplementary Material 2 (DOCX 36.9 KB)

Supplementary Material 3 (DOCX 349 KB)

## Data Availability

The datasets generated and/or analyzed during the current study are available from the corresponding authors on reasonable request.

## Abbreviations

1RM 1: Repetition Maximum
6MWT Six: Minute Walk Test
30s StS 30: second Sit-to-Stand
ABPM: Ambulatory Blood Pressure Monitoring
AHI: Apnea–Hypopnea Index
BMI: Body Mass Index
CPAP: Continuous Positive Airway Pressure
CPET: Cardiopulmonary Exercise Testing
DBP: Diastolic Blood Pressure
DLCO: Diffusing Capacity for Carbon Monoxide
EDS: Excessive Daytime Sleepiness
EMT: Expiratory Muscle Training
ESS: Epworth Sleepiness Scale
FEV₁: Forced Expiratory Volume in One Second
FIV₁: Forced Inspiratory Volume in One Second
FIVC: Forced Inspiratory Vital Capacity
FSS: Fatigue Severity Scale
FVC: Forced Vital Capacity
IMT: Inspiratory Muscle Training
MAP: Mean Arterial Pressure
MEP: Maximal Expiratory Pressure
MIP: Maximal Inspiratory Pressure
MoCA: Montreal Cognitive Assessment
MSNA: Muscle Sympathetic Nervous Activity
OSA: Obstructive Sleep Apnea
PEF: Peak Expiratory Flow
PICO: Population, Intervention, Comparison, and Outcomes
PIF: Peak Inspiratory Flow
PRISMA: Preferred Reporting Items for Systematic Reviews and Meta-Analyses
PROSPERO: International Prospective Register of Systematic Reviews
PSQI: Pittsburgh Sleep Quality Index
RCTs: Randomized Controlled Trials
RMT: Respiratory Muscle Training
RoB 2: Cochrane Risk of Bias 2.0
RQ: Respiratory Quotient
SBP: Systolic Blood Pressure
TLC: Total Lung Capacity
UA: Upper Airway
VE/BSA: Ratio of minute ventilation to body surface area
VE/MVV: Ratio between minute ventilation and maximal voluntary ventilation
WHO: World Health Organization

## References

[1] Mayoral LPC, Andrade GM, Mayoral EPC, Huerta TH, Canseco SP, Rodal Canales FJ et al (2020) Obesity subtypes, related biomarkers & heterogeneity. Indian J Med Res [Internet]. 1o de janeiro de 2020 [citado 13 de janeiro de 2026];151(1):11–21. Disponível em: https://pubmed.ncbi.nlm.nih.gov/32134010/32134010 10.4103/ijmr.IJMR_1768_17 PMC7055173

[2] World Health Organization (2025) Obesity and overweight [Internet]. [citado 8 de junho de 2025]. Disponível em: https://www.who.int/news-room/fact-sheets/detail/obesity-and-overweight

[3] Guglielmi G (2025) New obesity definition sidelines BMI to focus on health. Nat 1o de janeiro de 637(8047):773–774 10.1038/d41586-025-00123-1 39814926

[4] BrumM, Sturm R (2025) Severe obesity increases more rapidly in Brazil than moderate obesity: analysis of Vigitel 2006–2021. Revista Brasileira de Epidemiologia [Internet]. [citado 13 de janeiro de 2026];28:1–3. Disponível em: https://pubmed.ncbi.nlm.nih.gov/40136124/10.1590/1980-549720250011 PMC11932355 40136124

[5] Andrade A, dos Santos KM, D’Oliveira A, Claudino VM, da Cruz WM (2025) Physical activity as a protective factor in the mood of children and adolescents: association with overweight and obesity. Front pediatr [Internet]. [citado 13 de janeiro de 2026];13:1–7. Disponível em: https://pubmed.ncbi.nlm.nih.gov/40182006/10.3389/fped.2025.1494998 PMC11967400 40182006

[6] Destors M, Tamisier R, Galerneau LM, Lévy P, Pepin JL Physiopathologie du syndrome d’apnées-hypopnées obstructives du sommeil et de ses conséquences cardio-métaboliques. Presse medicale [Internet]. 1o de abril de 2017 [citado 17 de novembro de 2025];46(4):395–403. Disponível em: https://pubmed.ncbi.nlm.nih.gov/28126503/10.1016/j.lpm.2016.09.008 28126503

[7] McNicholas WT, Korkalainen H (2023) Translation of obstructive sleep apnea pathophysiology and phenotypes to personalized treatment: a narrative review. Front Neurol 14:1239016 24 de agosto de 2023 37693751 10.3389/fneur.2023.1239016 PMC10483231

[8] Moawd SA, Azab AR, Alrawaili SM, Abdelbasset WK (2020) Inspiratory muscle training in obstructive sleep apnea associating diabetic peripheral neuropathy: A randomized control study. Biomed Res Int [Internet]. [citado 13 de janeiro de 2026];2020:1–8. Disponível em: https://pubmed.ncbi.nlm.nih.gov/32626744/10.1155/2020/5036585 PMC7306097 32626744

[9] Pei Y, Fan Y, Kong X, Sun H, Zhou J, Wu H (2022) Investigation of the effectiveness of traditional breathing therapy on pulmonary function in college students with obstructive sleep apnea. Contrast media mol imaging [Internet]. [citado 13 de janeiro de 2026];2022:1674973. Disponível em: https://pmc.ncbi.nlm.nih.gov/articles/PMC9307394/10.1155/2022/1674973 PMC9307394 35909585

[10] Salzano G, Maglitto F, Bisogno A, Vaira LA, De Riu G, Cavaliere M et al (2021) Obstructive sleep apnoea/hypopnoea syndrome: Relationship with obesity and management in obese patients. Vol. 41, Acta Otorhinolaryngologica Italica. Pacini Editore S.p.A./AU-CNS; pp. 120–30 10.14639/0392-100X-N1100 PMC8142730 34028456

[11] Framnes SN, Arble DM, The bidirectional relationship between obstructive sleep apnea and metabolic disease. Front endocrinol (lausanne) [Internet]. 6 de agosto de 2018 [citado 17 de novembro de 2025];9(AUG). Disponível em: https://pubmed.ncbi.nlm.nih.gov/30127766/10.3389/fendo.2018.00440 PMC6087747 30127766

[12] Figorilli M, Velluzzi F, Redolfi S Obesity and sleep disorders: A bidirectional relationship. Nutrition, metabolism and cardiovascular diseases [internet]. 1o de junho de 2025 [citado 17 de novembro de 2025];35(6). Disponível em: https://pubmed.ncbi.nlm.nih.gov/40180826/10.1016/j.numecd.2025.104014 40180826

[13] Azeredo LM, de Souza LC, Guimarães BLS, Puga FP, Behrens NSCS, Lugon JR (2022) Inspiratory muscle training as adjuvant therapy in obstructive sleep apnea: a randomized controlled trial. Braz J Med Biol Res 55:1–7 10.1590/1414-431X2022e12331 PMC9529044 36197415

[14] Senaratna CV, Perret JL, Lodge CJ, Lowe AJ, Campbell BE, Matheson MC et al (2017) Prevalence of obstructive sleep apnea in the general population: A systematic review. Sleep Med Rev [Internet]. 1o de agosto de 2017 [citado 17 de novembro de 2025];34:70–81. Disponível em: https://pubmed.ncbi.nlm.nih.gov/27568340/27568340 10.1016/j.smrv.2016.07.002

[15] Benjafield AV et al (2019) Estimation of the global prevalence and burden of obstructive sleep apnoea: a literature-based analysis. Lancet Respir Med agosto de 7(8):687–698 10.1016/S2213-2600(19)30198-5 PMC7007763 31300334

[16] Salzano G, Maglitto F, Bisogno A, Vaira LA, De Riu G, Cavaliere M et al (2021) Obstructive sleep apnoea/hypopnoea syndrome: relationship with obesity and management in obese patients. Acta otorhinolaryngol ital [Internet]. 14 de maio de 2021 [citado 13 de janeiro de 2026];41(2):120–130. Disponível em: https://pubmed.ncbi.nlm.nih.gov/34028456/10.14639/0392-100X-N1100 PMC8142730 34028456

[17] de Sousa AS, da Rocha AP, Tavares DRB, Okazaki JÉF, Santana MV, de Pinto A (2022) Efetividade e segurança do treinamento muscular respiratório na apneia obstrutiva do sono: protocolo de uma revisão sistemática. Fisioterapia Brasil 22 de dezembro de 23(6):910–927

[18] Jehan S, Auguste E, Zizi F, Pandi-Perumal SR, Gupta R, Attarian H et al (2016) Obstructive sleep apnea: Women’s perspective. J sleep Med Disord [Internet] 3(6):1064 Disponível em:. https://pmc.ncbi.nlm.nih.gov/articles/PMC5323064/28239685 PMC5323064

[19] Dar JA, Mujaddadi A, Moiz JA (2022) Effects of inspiratory muscle training in patients with obstructive sleep apnoea syndrome: a systematic review and meta-analysis. Sleep Sci [Internet] 15(4):480–489 Disponível em:. https://pubmed.ncbi.nlm.nih.gov/36419804/36419804 10.5935/1984-0063.20220081 PMC9670769

[20] Weaver TE, Grunstein RR (2008) Adherence to continuous positive airway pressure therapy: The challenge to effective treatment. Vol. 5, Proceedings of the American Thoracic Society. pp. 173–8 10.1513/pats.200708-119MG PMC2645251 18250209

[21] Krause-Sorio B, An E, Aguila AP, Martinez F, Aysola RS, Macey PM (2021) Inspiratory muscle training for obstructive sleep apnea: Protocol development and feasibility of home practice by sedentary adults. Front Physiol. 4 de novembro de 2021;12:1–9 10.3389/fphys.2021.737493 PMC8599350 34803729

[22] Casagrande P, de O, Coimbra DR, de Souza LC, Andrade A (2023) Effects of yoga on depressive symptoms, anxiety, sleep quality, and mood in patients with rheumatic diseases: Systematic review and meta-analysis. Vol. 15, PM and R. John Wiley and Sons Inc; pp. 899–915 10.1002/pmrj.12867 35726183

[23] Al Oweidat K, Toubasi AA, Tawileh RBA, Tawileh HBA, Hasuneh MM (2023) Bariatric surgery and obstructive sleep apnea: a systematic review and meta-analysis. Sleep Breath [Internet]. 1o de dezembro de 2023 [citado 13 de janeiro de 2026];27(6):2283–2294. Disponível em: https://pubmed.ncbi.nlm.nih.gov/37145243/10.1007/s11325-023-02840-1 37145243

[24] Ashrafian H, Le Roux CW, Rowland SP, Ali M, Cummin AR, Darzi A et al (2012) Metabolic surgery and obstructive sleep apnoea: the protective effects of bariatric procedures. Thorax [Internet] 67(5):442–449 Disponível em:. https://pubmed.ncbi.nlm.nih.gov/21709167/21709167 10.1136/thx.2010.151225

[25] CamachoM, Certal V, Abdullatif J, Zaghi S, Ruoff CM, Capasso R et al (2015) Myofunctional therapy to treat obstructive sleep apnea: A systematic review and meta-analysis. Sleep [Internet]. 1o de maio de 2015 [citado 13 de janeiro de 2026];38(5):669–675. Disponível em: https://pubmed.ncbi.nlm.nih.gov/25348130/10.5665/sleep.4652 PMC4402674 25348130

[26] Qin H, Wang Y, Chen X, Steenbergen N, Penzel T, Zhang X et al The efficacy of bariatric surgery on pulmonary function and sleep architecture of patients with obstructive sleep apnea and co-morbid obesity: a systematic review and meta-analysis. Surg Obes Relat Dis [Internet]. 1o de dezembro de 2023 [citado 13 de janeiro de 2026];19(12):1444–57. Disponível em: https://pubmed.ncbi.nlm.nih.gov/37673709/10.1016/j.soard.2023.07.007 37673709

[27] Srijithesh PR, Aghoram R, Goel A, Dhanya J Positional therapy for obstructive sleep apnoea. Cochrane Database Syst Rev [Internet]. 1o de maio de 2019 [citado 13 de janeiro de 2026];5(5). Disponível em: https://pubmed.ncbi.nlm.nih.gov/31041813/10.1002/14651858.CD010990.pub2 PMC6491901 31041813

[28] Lin HC, Chiang LL, Ong JH, Tsai K, ling, Hung CH, Lin CY (2020) The effects of threshold inspiratory muscle training in patients with obstructive sleep apnea: a randomized experimental study. Sleep and Breathing [Internet]. 1o de março de 2020 [citado 13 de janeiro de 2026];24(1):201–209. Disponível em: https://tcu.elsevierpure.com/en/publications/the-effects-of-threshold-inspiratory-muscle-training-in-patients-/31115739 10.1007/s11325-019-01862-y

[29] Liu S, Fan Z, FU M, Cheng K, Zhang X, Ni J et al (2025) Impact of inspiratory muscle training on aspiration symptoms in patients with dysphagia following ischemic stroke. Brain Res. 1o de março de 2025;1850:149396 10.1016/j.brainres.2024.149396 39662789

[30] Page MJ, Moher D, Bossuyt PM, Boutron I, Hoffmann TC, Mulrow CD et al (2021) PRISMA 2020 explanation and elaboration: updated guidance and exemplars for reporting systematic reviews. BMJ [Internet] 29 de março de 2021 [citado 14 de outubro de 2021];372: n160. Disponível em: https://www.bmj.com/content/372/bmj.n160 10.1136/bmj.n160 PMC8005925 33781993

[31] Ouzzani M, Hammady H, Fedorowicz Z, Elmagarmid A (2016) Rayyan-a web and mobile app for systematic reviews. Syst Rev 5 de dezembro de;5(1):210 10.1186/s13643-016-0384-4 PMC5139140 27919275

[32] Shiwa RS, Oliveira LPC, Moser AD, de Aguiar L, de Oliveira I (2011) LVF de. PEDro: a base de dados de evidências em fisioterapia. Fisioter Mov. ;24(3):523–33

[33] HigginsJ, Thomas J, Chandler (2023) Cochrane handbook for systematic reviews of interventions version 6.4 (updated August 2023). Cochrane 2024:1–925 Disponível em: www.cochrane.org/handbook https://www.cochrane.org/handbook

[34] SterneJAC,Savović J, Page MJ, Elbers RG, Blencowe NS, Boutron I et al (2019) RoB 2: a revised tool for assessing risk of bias in randomised trials. The BMJ [Internet]. 28 de agosto de 2019 [citado 27 de agosto de 2024];366: l4898. Disponível em: https://www.bmj.com/content/366/bmj.l4898 10.1136/bmj.l4898 31462531

[35] Andhare NM, Yeole U, Fakhri F Comparison of inspiratory muscle training & conventional breathing exercises in obstructive sleep apnoea: A randomized control trial. UGC Care Journal [Internet]. 5 de abril de 2020 [citado 13 de janeiro de 2026];19(5):461–8. Disponível em: https://www.researchgate.net/publication/343650319_Comparison_of_Inspiratory_Muscle_Training_Conventional_Breathing_Exercises_in_Obstructive_Sleep_Apnoea_A_Randomized_Control_Trial

[36] Mannarino MR, Di Filippo F, Pirro M (2012) Obstructive sleep apnea syndrome. European Journal of Internal Medicine, vol 23. Elsevier B.V., pp 586–593 10.1016/j.ejim.2012.05.013 22939801

[37] Nóbrega-Júnior JCN, de Andrade AD, de Andrade EAM, Andrade MDA, Ribeiro ASV, Pedrosa RP et al (2020) Inspiratory muscle training in the severity of obstructive sleep apnea, sleep quality and excessive daytime sleepiness: A placebo-controlled, randomized trial. Nat Sci Sleep [Internet]. 1o de dezembro de 2020 [citado 13 de janeiro de 2026];12:1105. Disponível em: https://pmc.ncbi.nlm.nih.gov/articles/PMC7719323/33293881 10.2147/NSS.S269360 PMC7719323

[38] Erturk N, Calik-Kutukcu E, Arikan H, Savci S, Inal-Ince D, Caliskan H et al (2020) The effectiveness of oropharyngeal exercises compared to inspiratory muscle training in obstructive sleep apnea: A randomized controlled trial. Heart Lung [Internet]. 1o de novembro de 2020 [citado 13 de janeiro de 2026];49(6):940–948. Disponível em: https://www.heartandlung.org/action/showFullText?pii=S0147956320303149 32800391 10.1016/j.hrtlng.2020.07.014

[39] Ramos-BarreraGE, DeLucia CM, Bailey EF (2020) Inspiratory muscle strength training lowers blood pressure and sympathetic activity in older adults with OSA: a randomized controlled pilot trial. Journal of Applied Physiology [Internet]. 1o de setembro de 2020 [citado 13 de janeiro de 2026];129(3):449. Disponível em: https://pmc.ncbi.nlm.nih.gov/articles/PMC10205163/32730174 10.1152/japplphysiol.00024.2020 PMC10205163

[40] Stavrou VT, Vavougyios GD, Tsirimona G, Boutlas S, Santo M, Hadjigeorgiou G et al (2076) The effects of 4-week respiratory muscle training on cardiopulmonary parameters and cognitive function in male patients with OSA. Applied Sciences 2025, Vol 15, [Internet]. 25 de fevereiro de 2025 [citado 13 de janeiro de 2026];15(5). Disponível em: https://www.mdpi.com/ -3417/15/5/2532

[41] Ferreira STBP, do Socorro Brasileiro-Santos M, Teixeira JB, da Silva Rabello MC, de Lorena VMB, Farah BQ et al Clinical safety and hemodynamic, cardiac autonomic and inflammatory responses to a single session of inspiratory muscle training in obstructive sleep apnea. Sleep and Breathing 2021 26:1 [Internet]. 5 de abril de 2021 [citado 13 de janeiro de 2026];26(1):99–108. Disponível em: https://link.springer.com/10.1007/s11325-021-02364-6 10.1007/s11325-021-02364-6 33821439

[42] Vaz AP, Drummond M, Mota PC, Severo M, Almeida J, Winck JC Translation of Berlin Questionnaire to Portuguese language and its application in OSA identification in a sleep disordered breathing clinic. Revista Portuguesa de Pneumologia (English Edition) [Internet]. 30 de março de 2011 [citado 13 de janeiro de 2026];17(2):59–65. Disponível em: https://www.tandfonline.com/doi/pdf/10.1016/S2173-5115(11)70015-X 21477567

[43] Montemezzo D, Velloso M, Rodrigues Britto R, Parreira VF, Verônica P, Parreira F Pressões respiratórias máximas: equipamentos e procedimentos usados por fisioterapeutas brasileiros. Fisioterapia e Pesquisa [Internet]. junho de 2010 [citado 13 de janeiro de 2026];17(2):147–152. Disponível em: https://www.scielo.br/j/fp/a/QBGtwyztRCGYdtGbbxbL34L/?format=html=pt

[44] Silva PE, de Carvalho KL, Frazão M, Maldaner V, Daniel CR, Gomes-Neto M Assessment of maximum dynamic inspiratory pressure. Respir Care [Internet]. 1o de outubro de 2018 [citado 13 de janeiro de 2026];63(10):1231–1238. Disponível em: https://scholar.google.com/scholar_url?url=https://journals.sagepub.com/doi/pdf/10.4187/respcare.06058&hl=pt-BR&sa=T&oi=ucasa&ct=ufr&ei=7rlnaePOFcelieoPl7ztyQY&scisig=AHkA5jRXwXxp4ICRQb7ljQZixzBD 10.4187/respcare.06058 30018174

[45] Holland AE, Spruit MA, Singh SJ How to carry out a field walking test in chronic respiratory disease. Breathe (Sheff) [Internet]. 1o de junho de 2015 [citado 13 de janeiro de 2026];11(2):129–39. Disponível em: https://pubmed.ncbi.nlm.nih.gov/26306113/10.1183/20734735.021314 PMC4487379 26306113

[46] Ozcan Kahraman B, Ozsoy I, Akdeniz B, Ozpelit E, Sevinc C, Acar S et al (2020) Test-retest reliability and validity of the timed up and go test and 30-second sit to stand test in patients with pulmonary hypertension. Int J Cardiol [Internet]. 1o de abril de 2020 [citado 13 de janeiro de 2026];304:159–163. Disponível em: https://pubmed.ncbi.nlm.nih.gov/31980271/31980271 10.1016/j.ijcard.2020.01.028

[47] de Albuquerque ALP, Berton DC, Ferreira Álvares S, Campos EVM, Queiroga-Júnior FJP, Santana ANC, de Wong B (2024) MS, New spirometry recommendations from the Brazilian Thoracic Association – 2024 update. Jornal Brasileiro de Pneumologia. ;50(6):e20240169 10.36416/1806-3756/e20240169 PMC11796395 39841775

[48] Máximo LSN, Melo FF, Porto LB, da Silva ICR, Do Prado M, Pedrosa HC (2019) Correlation of body composition parameters using different methods among brazilian obese adults. Revista Brasileira de Cineantropometria e Desempenho Humano.21:e60539

[49] DeMers D, Wachs D, Physiology Mean Arterial Pressure. StatPearls [Internet]. 10 de abril de 2023 [citado 13 de janeiro de 2026]; Disponível em: https://www.ncbi.nlm.nih.gov/books/NBK538226/

[50] Asayama K, Fujiwara T, Hoshide S, Ohkubo T, Kario K, Stergiou GS et al (2019) Nocturnal blood pressuremeasured by home devices: Evidence and perspective for clinical application. J Hypertens [Internet]. 1o de maio de 2019 [citado 13 de janeiro de 2026];37(5):905–916. Disponível em: https://journals.lww.com/jhypertension/fulltext/2019/05000/nocturnal_blood_pressure_measured_by_home_devices_.6.aspx 30394982 10.1097/HJH.0000000000001987

[51] Caminal P, Sola F, Gomis P, Guasch E, Perera A, Soriano N et al Validity of the Polar V800 monitor for measuring heart rate variability in mountain running route conditions. Eur J Appl Physiol 2018 118:3 [Internet]. 22 de janeiro de 2018 [citado 13 de janeiro de 2026];118(3):669–677. Disponível em: https://link.springer.com/10.1007/s00421-018-3808-0 10.1007/s00421-018-3808-0 29356949

[52] Bertolazi AN, Fagondes SC, Hoff LS, Pedro VD, Barreto SSM, Johns MW (2009) Portuguese-language version of the Epworth sleepiness scale: validation for use in Brazil. J Bras Pneumol [Internet] 35(9):877–883 Disponível em:. https://pubmed.ncbi.nlm.nih.gov/19820814/19820814 10.1590/s1806-37132009000900009

[53] Won Park K, Wook Kim H, Rye Choi M, Jo Kim B, Hyung Kim T, Sun Song O et al (2017) The effects of sleep apnea and variables on cognitive function and the mediating effect of depression. 24(2):86–96 Disponível em:. 10.14401/KASMED.2017.24.2.86. Sleep Medicine and Psychophysiology [Internet]

[54] Chiu HY, Chen PY, Chuang LP, Chen NH, Tu YK, Hsieh YJ et al (2017) Diagnostic accuracy of the Berlin questionnaire, STOP-BANG, STOP, and Epworth sleepiness scale in detecting obstructive sleep apnea: A bivariate meta-analysis. Sleep Med Rev [Internet]. 1o de dezembro de 2017 [citado 15 de janeiro de 2026];36:57–70. Disponível em: https://www.sciencedirect.com/science/article/pii/S1087079216301277 27919588 10.1016/j.smrv.2016.10.004

[55] LvR, Liu X, Zhang Y, Dong N, Wang X, He Y et al (2023) Pathophysiological mechanisms and therapeutic approaches in obstructive sleep apnea syndrome. Signal Transduction and Targeted Therapy [Internet]. 1o de dezembro de 2023 [citado 13 de janeiro de 2026];8(1):218. Disponível em: https://pmc.ncbi.nlm.nih.gov/articles/PMC10211313/10.1038/s41392-023-01496-3 PMC10211313 37230968

[56] Zimberg IZ, de Melo CM, Del RM, dos Santos MV, Crispim CA, Lopes T, do VC et al Relação entre apneia obstrutiva do sono e obesidade: uma revisão sobre aspectos endócrinos, metabólicos e nutricionais. RBONE - Revista Brasileira de Obesidade, Nutrição e Emagrecimento [Internet]. 18 de junho de 2017 [citado 13 de janeiro de 2026];11(64):250–60. Disponível em: https://www.rbone.com.br/index.php/rbone/article/view/527

[57] Caicedo-Trujillo S, Torres-Castro R, Vasconcello-Castillo L, Solis-Navarro L, Sanchez-Ramirez D, Núñez-Cortés R et al (2023) Inspiratory muscle training in patients with obesity: a systematic review and meta-analysis. Frontiers in medicine, vol 10. Frontiers Media SA 10.3389/fmed.2023.1284689 PMC10711597 38089877

[58] Vivodtzev I, Tamisier R, Croteau M, Borel JC, Grangier A, Wuyam B et al (2018) Ventilatory support or respiratory muscle training as adjuncts to exercise in obese CPAP-treated patients with obstructive sleep apnoea: a randomised controlled trial. Thorax [Internet] 1o de julho de 2018 [citado 13 de janeiro de 2026];73(7):634–643. Disponível em: https://pubmed.ncbi.nlm.nih.gov/29463621/10.1136/thoraxjnl-2017-211152 29463621

[59] Souza AKF, Dornelas de Andrade A, de Medeiros AIC, de Aguiar MIR, Rocha TD, de Pedrosa S RP (2018) Effectiveness of inspiratory muscle training on sleep and functional capacity to exercise in obstructive sleep apnea: a randomized controlled trial. Sleep Breath [Internet]. 1o de setembro de 2018 [citado 13 de janeiro de 2026];22(3):631–639. Disponível em: https://pubmed.ncbi.nlm.nih.gov/29124630/29124630 10.1007/s11325-017-1591-5

[60] Ayik S, Dalli A, Sözmen Mkakhan G (2024) Evaluation of airway responsiveness and pulmonary function test results among obese and non-obese patients with obstructive sleep apnea syndrome. European review for medical and pharmacological sciences. abril de 2024; 28(8):3056–3065 10.26355/eurrev_202404_36021 38708464

[61] Mello PR, Guerra GM, Borile S, Rondon MU, Alves MJ, Negrão CE et al Inspiratory muscle training reduces sympathetic nervous activity and improves inspiratory muscle weakness and quality of life in patients with chronic heart failure: a clinical trial. J Cardiopulm Rehabil Prev [Internet]. setembro de 2012 [citado 13 de janeiro de 2026];32(5):255–61. Disponível em: https://pubmed.ncbi.nlm.nih.gov/22785143/10.1097/HCR.0b013e31825828da 22785143

[62] Zheng SQ, Zhang Q, Li SY, Li S, Yao Q, Zheng X et al (2023) Effects of inspiratory muscle training in patients with hypertension: a meta-analysis. Front Cardiovasc Med [Internet]. 23 de maio de 2023 [citado 13 de janeiro de 2026]; 10:1113509. Disponível em: https://www.frontiersin.org/journals/cardiovascular-medicine/articles/10.3389/fcvm.2023.1113509/full 10.3389/fcvm.2023.1113509 PMC10270119 37332584

[63] Ieto V, Kayamori F, Montes MI, Hirata RP, Gregório MG, Alencar AM et al Effects of Oropharyngeal Exercises on Snoring: A Randomized Trial. Chest [Internet]. 1o de setembro de 2015 [citado 13 de janeiro de 2026];148(3):683–691. Disponível em: https://pubmed.ncbi.nlm.nih.gov/25950418/10.1378/chest.14-2953 25950418

