# Supplementary material for: Effects of inspiratory muscle training in adults with obesity and obstructive sleep apnea: a systematic review
Source: Sleep Breath. 2026 Apr 25;30(2):141. doi: 10.1007/s11325-026-03689-w (PMC13110206; doi:10.1007/s11325-026-03689-w)

**Article title:** Effects of Inspiratory Muscle Training in adults with obesity and obstructive sleep apnea: A systematic review

**Journal name:** [Sleep and Breathing](https://link.springer.com/journal/11325)

**Author names:** Karina Abreu^1*^, Amanda Farias e Farias^1^, Ananda Quaresma Nascimento^1^, Alexandro Andrade^1^ and Darlan Laurício Matte^1*^

**Affiliation:** 1 Center for Health and Sports Sciences – CEFID/Santa Catarina State University (UDESC), Florianópolis, Santa Catarina, Brasil.

**Corresponding authors. E-mails:** fisioterapeutakarina.abreu@gmail.com; darlan.matte.phd@gmail.com.

**Supplementary Information (SI)**

**Supplement 1** Methodological quality of studies assessed by the Pedro Scale. Y (presents the recommended criteria); N (does not present the recommended criteria); Criteria: 2 (Random allocation); 3 (Concealed allocation); 4 (Basal comparability); 5 (Blind subjects); 6 (Blind therapists); 7 (Blind evaluators); 8 (Adequate follow-up); 9 (Intention-to-treat analysis); 10 (Comparisons between groups); 11 (Point estimates and variability).


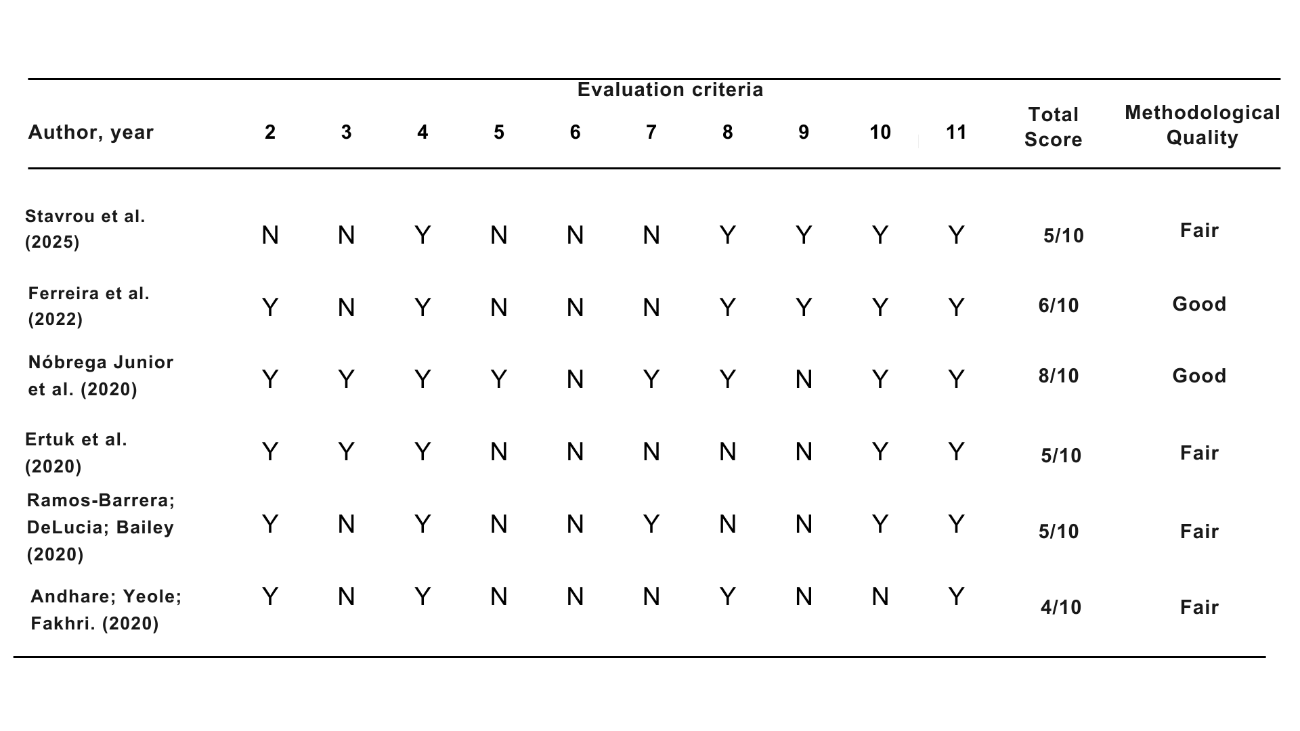

Supplement: Supplementary file 1 — Supplementary Material 1 (DOCX 53.5 KB) [file 11325_2026_3689_MOESM1_ESM.docx]
