# Supplementary material for: Effects of inspiratory muscle training in adults with obesity and obstructive sleep apnea: a systematic review
Source: Sleep Breath. 2026 Apr 25;30(2):141. doi: 10.1007/s11325-026-03689-w (PMC13110206; doi:10.1007/s11325-026-03689-w)

**Article title:** Effects of Inspiratory Muscle Training in adults with obesity and obstructive sleep apnea: A systematic review

**Journal name:** [Sleep and Breathing](https://link.springer.com/journal/11325)

**Author names:** Karina Abreu^1*^, Amanda Farias e Farias^1^, Ananda Quaresma Nascimento^1^, Alexandro Andrade^1^ and Darlan Laurício Matte^1*^

**Affiliation:** 1 Center for Health and Sports Sciences – CEFID/Santa Catarina State University (UDESC), Florianópolis, Santa Catarina, Brasil.

**Corresponding authors. E-mails:** fisioterapeutakarina.abreu@gmail.com; darlan.matte.phd@gmail.com.

**Supplementary Information (SI)**

**Supplement 2** Risk of bias of the studies assessed by Cochrane Risk of Bias 2. D1 (Randomization process); D2 (Deviations from the intended interventions); D3 (Missing outcome data); D4 (Measurement of the outcome); D5 (Selection of the reported result).


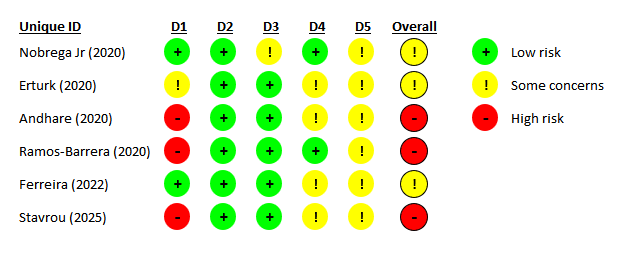

Supplement: Supplementary file 2 — Supplementary Material 2 (DOCX 36.9 KB) [file 11325_2026_3689_MOESM2_ESM.docx]
