# Supplementary material for: Effects of inspiratory muscle training in adults with obesity and obstructive sleep apnea: a systematic review
Source: Sleep Breath. 2026 Apr 25;30(2):141. doi: 10.1007/s11325-026-03689-w (PMC13110206; doi:10.1007/s11325-026-03689-w)
Supplement: Supplementary file 3 — Supplementary Material 3 (DOCX 349 KB) [file 11325_2026_3689_MOESM3_ESM.docx]

**Article title:** Effects of Inspiratory Muscle Training in adults with obesity and obstructive sleep apnea: A systematic review

**Journal name:** [Sleep and Breathing](https://link.springer.com/journal/11325)

**Author names:** Karina Abreu^1*^, Amanda Farias e Farias^1^, Ananda Quaresma Nascimento^1^, Alexandro Andrade^1^ and Darlan Laurício Matte^1*^

**Affiliation:** 1 Center for Health and Sports Sciences – CEFID/Santa Catarina State University (UDESC), Florianópolis, Santa Catarina, Brasil.

**Corresponding authors. E-mails:** fisioterapeutakarina.abreu@gmail.com; darlan.matte.phd@gmail.com.

**Supplementary Information (SI)**

Supplement 3: Schematic representation of the effects of IMT in individuals with obesity and OSA.

**
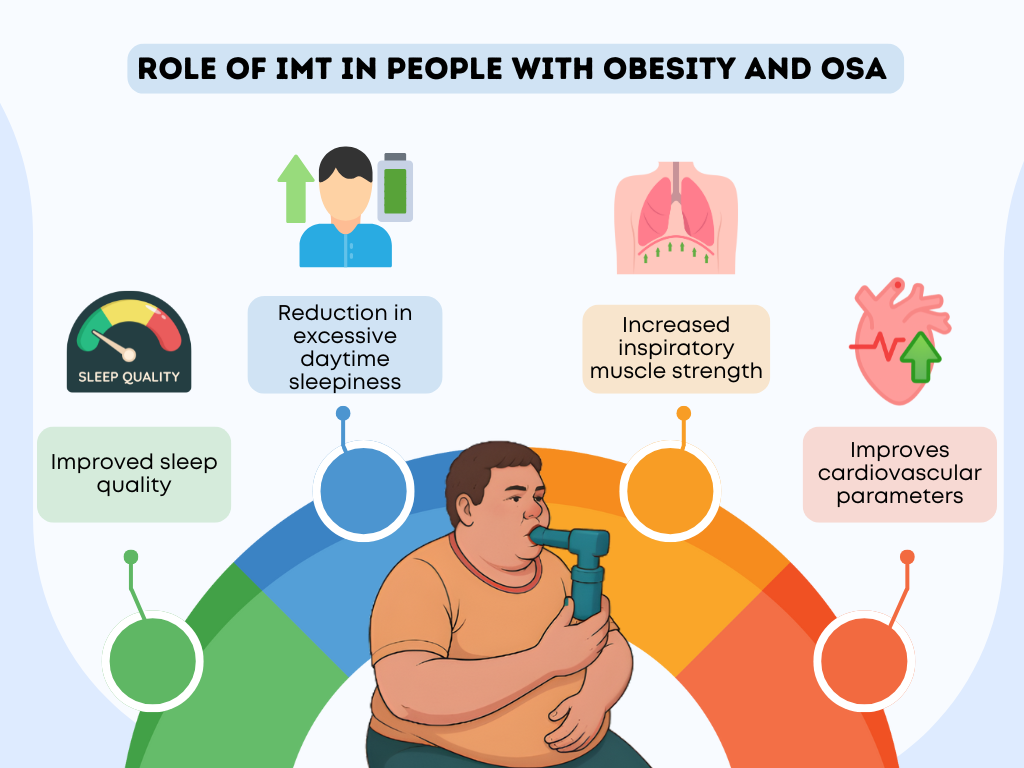
**
